# Pericardiectomy in post-traumatic suppurative constrictive pericarditis: case report

**DOI:** 10.1186/1757-1626-2-9292

**Published:** 2009-12-09

**Authors:** Abiodun Oyinpreye Jasper, Gandhi Agbaegbe Anyanhun, Stanley Ukadike Okugbo, Philomena Adudu

**Affiliations:** 1 Department of Anaesthesia, College of Health Sciences, Delta State University, Abraka Delta State, Nigeria; 2 Department of Surgery, University of Benin Teaching Hospital, Benin, Nigeria; 3 Department of Surgery, University of Benin Teaching Hospital, Benin, Edo State, Nigeria; 4 Department of Anaesthesia, University of Benin Teaching Hospital, Benin, Edo State, Nigeria

## Abstract

A 13-year-old male was seen at the Hospital with a 5-months history of right chest swelling, pain and recurrent fever and breathlessness on mild exertion. There was a history of gunshot to his chest two and half years before presentation.

On admission he was febrile with a temperature of 39°C. The chest wall swelling measuring 6 cm/6 cm was tender and fluctuant and needle aspiration yielded purulent fluids. His blood pressure and pulse were 110/60 mmHg (14.6/8 Kpa) and 100 per minute respectively. The chest radiograph showed massive cardiomegaly with pellets lodging in the right 5^th ^costosternal joint. Echocardiography confirmed massive pericardial effusion. The electrocardiogram showed sinus rhythm and low voltage of QRS complexes without chamber enlargement. A diagnosis of constrictive pericarditis with purulent pericardial effusion secondary to foreign body abscess was made.

His clinical picture improved after an initial incision and drainage of the right anterior chest wall abscess under ketamine intravenous anaesthesia. Two weeks after, he had pericardiectomy under general anaesthesia using a nitrous/oxygen/halothane relaxant technique which was uneventful.

## Introduction

Penetrating chest wall injury is a known cause of pericarditis though viral pericarditis following an upper respiratory infection is perhaps the commonest cause of acute pericarditis in temperate climates. In the tropics acute suppurative pericarditis follows inadequately treated bacterial pneumonias. Mycobacteria tuberculosis as a cause of pericarditis occurs as a direct extension of pulmonary tuberculosis increasingly associated with human immunoglobulin deficiency syndrome (HIV-AIDS) in sub-Saharan Africa [1]

This case illustrates the anaesthetic management of a patient with post-traumatic suppurative pericardial effusion and chest wall involvement for pericardiectomy.

## Case presentation

A 13-year-old male of Yoruba, Nigerian descent weighing 30 kg was referred to the cardiothoracic unit of the University of Benin Teaching Hospital with a five-month history of chest swelling, chest pain, recurrent fever and breathlessness on mild exertion. He was shot in the chest two years earlier by armed robbers. Though not fatal, bullet pellets were left in chest wall after treatment in a private hospital. Past medical history was not significant.

Physical examination revealed a febrile (temperature 39°C) and ill-looking boy with palpably enlarged axillary and supraclavicular lymph nodes. His jugular venous pressure was raised to the angle of the jaw. The right chest swelling measuring 6/6 cm was tender and fluctuant and needle aspiration yielded purulent fluids. Breath sounds were diminished on the right lung field. The pulse rate and blood pressure were 100 beats per minute and 110/60 mmHg (14.6/8 Kpa) respectively. On auscultation the heart sounds I, II were distant and no murmurs were heard. He was classified as class 3 according to the New York Heart Association functional classification.

A chest radiograph revealed massive cardiomegaly with pellets logging in the right 5^th ^costosternal joint anterior chest wall (on postero-anterior view) and features of pleural effusion. Echocardiography confirmed pericardial effusion; and a diagnosis of constrictive pericarditis with suppurative pericardial effusion was made. His packed cell volume (PCV) was 22% (haemoglobin concentration of 7 gm/dl) Human immunoglobulin virus (HIV) and Mantoux tests were negative. The ECG revealed sinus rhythm of 100 beats per minute, low voltage QRS complexes. Total serum protein was also low (8.3 gdL^-1^) Patient was transfused with a unit of blood and his haemoglobin rose to 11 gdL^-1^.

On preoperative visit, there was a history of previous anaesthesia and surgery, for incision and drainage of chest wall abscess under intravenous ketamine anaesthesia and oxygen by face mask, two weeks earlier. This was eventful. Based on clinical findings of dyspnoea on mild exertion raised jugular venous pressure and tachycardia suggesting constrictive pericarditis, he was graded as ASA physical status 3 and class 2 according to New York Heart Association functional classification. For premedication he had oral diazepam 5 mg in the night and morning of surgery respectively.

Baseline pulse and blood pressure were 100 beats per minute and 120/80 mmHg (16/10.6 Kpa) respectively. The ECG showed sinus rhythm with low voltage of QRS complexes. Venous access was obtained and he was pre-oxygenated with 100 percent oxygen for three minutes. Intravenous 1% lignocaine, 30 mg was given prior to induction of anaesthesia with 5 mg midazolam. Laryngoscopy and intubation were facilitated with 30 mg of suxamethonium i.v. using a size 6.5 mm cuffed polyvinyl chloride endotracheal tube. A total dose of 10 mg i.v pancuronium bromide was used for muscle relaxation. Intermittent positive pressure ventilation was instituted using a flow rate of 6 litres/min (3 1/min O_2 _and 3 1/min of N_2_O). Analgesia was achieved with 15 mg of pentazocine i.v up to a total dose of 30 mg for the procedure.

The pulse, blood pressure, ECG, peripheral and core (nasal cavity) temperatures and urine output were monitored at regular intervals. The pulse rate and blood pressure ranged between 100 - 110 beats per minutes and 100/60 mmHg to 120/70 mmHg respectively. The peripheral and core temperature ranged between 36.8°C and 36.5°C and between 18.5 and 13 mmH20. Blood loss was estimated at 300 ml. Fluid maintenance was with 2.5 litres of crystalloids.

During surgery, the patient developed ventricular ectopic beats, which was treated with 30 mg of intravenous 1% lignocaine. A chest tube drain was connected to an underwater seal drainage system. The urine output during the five-hour surgery was 1000 ml.

At the end of the surgery, residual muscle paralysis was reversed with intravenous neostigmine methylsulphate; 1.2 mg and atropine sulphate 0.6 mg. After extubation, he was given 8 L/minute of oxygen by a facemask for about 5 minutes and was transferred to the recovery room. He had ventricular ectopic beats that responded to intravenous lignocaine, anaesthesia. He was observed for about 45 minutes in the recovery room while on 5 L/minute of oxygen by a polymask. He was then transferred to the intensive care unit (ICU).

In the ICU he was nursed in the cardiac position with humidified oxygen by a face mask at the rate of 5 litres per minute for the first 24 hours. His ICU stay was quite uneventful. Post-operative haemoglobin level was 10 g/dL.

## Discussion

Acute pericarditis could be serous, purulent or haemorrhagic depending upon the cause. The exudates compress the underlying myocardium limiting cardiac distension during diastole. Venous return to the heart is consequently diminished. Ventricular contraction is also diminished and cardiac output is reduced [1]. Constrictive pericarditis results when the healing of an acute fibrinous or sero-fibrinous pericarditis is followed by obliteration of the pericardial cavity, with the formation of granulation tissue, which gradually contracts and forms a firm scar encasing the heart and interfering with filling. Purulent pericarditis is rare and may be occur as a complication of septicaemia, direct spread from intrathoracic infection or from a penetrating injury [1].

Early complaints of constrictive pericarditis are shortness of breath on exertion, recurrent chest pain and or symptoms of right-sided heart failure with venous congestion, hepatomegaly, ascites and oedema [2]. Patient presented with complaints of severe breathlessness on mild exertion and the right chest wall swelling. Dyspnoea is an important and usually progressive symptom and is graded according to the New York Heart Association (NYHA) scale. The patient reported was classified as III according to NYHA on admission. NYHA classification was also used to assess the functional status of surviving patients in the group studied by DeValeria. About 87% were in either NYHA class I or II and had improved functional capacity at follow up[3]. In another study done by Pedreira Perez and colleagues Tuberculosis was the most frequent cause (68.3%) followed by idiopathic cases (24.1%). Preoperatively 3.4% were in New York Heart Association Class I, 31% in Class II, 48.3% in Class III and 17.2% in Class IV[4]. A history of malignancy, previous pericardial procedure, and preoperative New York Heart Association class IV were found to be predictors of poor survival [3].

The chest radiograph of the patient reported revealed cardiomegaly and pericardial effusions. His electrocardiogram showed sinus rhythm with a heart rate of 100 per minute and low voltage of QRS complexes. In addition, the echocardiograph showed pericardial effusions with reduced contraction of the ventricles. Based on the clinical findings and investigations the diagnosis of constrictive pericarditis was confirmed. Echocardiography is the definitive investigation for the detection of pericardial effusion since it is sensitive, specific, simple, innocuous and non invasive, and may be performed at the bedside. Echocardiography can detect effusions as small as 20 ml [1].

Pericardiocentesis is the management of pericardial effusion. The drainage of the right chest will abscess two weeks earlier under ketamine anaesthesia relieved the tamponade effect of the purulent pericarditis; suggesting a fistulous connection between the foreign abscess and the purulent pericarditis. The indications for pericardiectomy are persistently enlarged heart or progressive congestive heart failure, reduction in heart size with a rising venous pressure [5].

Anaesthesia for patients with constrictive pericarditis poses considerable challenges to the anaesthetist. They include a fixed cardiac output state and an inability of the heart to respond appropriately to stresses of surgery and anaesthesia. Also the presence of complications, which increased morbidity in this patient, included cardiomegaly and pleura effusion. His physical status was ASA III and it has been shown that ASA classification has a strong correlation with intraoperative and postoperative complications. During the preoperative assessment, the anaesthetist must determine if the patient is at risk so that appropriate interventions can be made to ensure the best possible outcome. The anaesthetists findings may not only affect the choice and technique of anaesthesia, but may also indicate likely and often serious complications during and after the operation. The most important considerations are the nature and severity of the cardiac lesion and its effect on the cardio- respiratory functional state [6]. Pedriera also noted that there were 4 in-hospital deaths (overall operative mortality 6.89%). Operative mortality in ten years was 0%. Low output was the most common nonfatal complication of pericardiectomy (15.5%). Mortality per patient year was 2.04%. Actuarial survival estimates were 82% and 71% at 5 to 10 years respectively. Postoperatively 76% were in New York Heart Association Class I, 16% in Class II, 8% in Class III and none in Class IV [4].

The incidence of perioperative arrhythmias is high in patients with cardiac diseases. Anaesthesia involves the use of a variety of drugs, some of which have either anti-arrhythmic or pro-arrhythmic potential. The marked alternations in autonomic nervous system activity associated with both anaesthesia and surgery, suggests that the perioperative period should be regarded as essentially pro-arrhythmic [7]. Alterations in blood gases, acid-base status and electrolytes, the effects of numerous drugs used in anaesthesia, and the autonomic consequences of anaesthesia and surgical stimulation may all influence the development of arrhythmias. It is clearly logical to minimize the pro-arrhythmic effects of surgery by properly conducted, balanced anaesthesia. Intravenous lignocaine; 50 mg of 1% was given to this patient prior to induction to attenuate haemodynamic response to laryngoscopy, and intubation. Tachycardia will lead to increase oxygen demand with reduction in blood supply because of the shorter duration of diastole, which reduces coronary blood flow. There is therefore need to control blood pressure response to laryngoscopy and intubation. The problem is particularly marked in patients with cardiac diseases. Numerous techniques have been employed in order to attenuate these haemodynamic responses including topical and intravenous lignocaine, vasodilators, opioids and adrenoceptor blockers.

The anaesthetic agents used most often has been fentanyl and diazepam with nitrous oxide, oxygen and pancuronium. Any anaesthetic agent that does not markedly decrease venous return or depress the myocardium can be used [8]. Midazolam was used for induction of anaesthesia in the case reported because it is cardiostable [9]. Some workers have suggested that inhalation agents or intravenous agents can be used, provided that great care is taken to limit total dosage and rate of administration [10]. It is important to minimize use of drugs and manipulations that decrease venous return, reduce heart rate, and cause hypertension, resulting in hypoxaemia or impairment ventricular contractility. Particular attention was paid to the mode of ventilation; as positive pressure ventilation, further embarrasses venous return and cardiac output. This brings out the wisdom in adopting spontaneous ventilation in the earlier incision and drainage of the chest wall abscess under ketamine anaesthesia 2 weeks before Pericardiectomy thereby increasing his chance of survival. The relief of tamponade effect informed the use of IPPV for pericardiectomy.

Electrocardiographic monitoring is mandatory during cardiac surgery and it has been discovered that the unipolar leads (VI-V6) may be superior to the traditional lead II in early detection of perioperative ischaemia. The rate pressure product (RPP- heart rate × systolic arterial pressure) is a useful clinical measurement of oxygen balance. The simple index may predict the point at which ischaemia occurs in the conscious exercising patient. During anaesthesia RPP should be maintained below the conscious ischaemic value; especially at tracheal intubation or extubation [11]. Central venous pressure monitoring (though not done in this patient)is essential during pericardiectomy both for the maintenance of right ventricular filling pressure and also to observe the fall in central venous pressure that follows the surgical relief of the constriction. Besides an ECG, a continuous display of arterial pressure is also useful. Other monitors of immense value in this patient were: pulse oximetry, temperature, urine output, and sphygmomanometer and pulse measurement. Pericardiectomy is the removal of both parietal pericardium and visceral peel from phrenic nerve to phrenic nerve. The selection of incision and the use of cardiopulmonary bypass have various proponents. Use of a median sternotomy with the aid of cardiopulmonary bypass for constrictive pericarditis has been reported to provide good results. Conors and co-workers [12]. advocated a left anterior thoracotomy for effusive disease. Culliford and colleagues [13]. reported that a median sternotomy was associated with the shortest hospitalization of patients undergoing Pericardiectomy for constrictive pericarditis. Regardless of the surgical approach or use of cardiopulmonary bypass (CPB), investigators have reported normalization of cardiac haemodynamics after radical Pericardiectomy [14].

Postoperative care in the ICU included maintenance of a semi-upright (> 30 degrees) position, supplemental oxygen (40 - 50%), close electrocardiographic and hemodynamic monitoring care of the chest tube and aggressive pain relief. Pain can be severe after an open thorax procedure, and adequacy in the postoperative thoracic surgery patient is of high importance. Pain also results in an increase in sympathetic nervous system activities, which is manifested by tachycardia, hypertension, and increased oxygen consumption; this latter condition predisposes the patient to myocardial ischemia. The administration of intramuscular pentazocine however proved adequate for postoperative analgesia in this patient.

## Conclusion

Complete pericardiectomy using a right anterolateral thoracotomy can be performed safely and can lead to long-term survival and good functional results. The careful preoperative assessment of patients with cardiovascular diseases makes it possible to establish the correct diagnosis, to evaluate the functional reserve of the heart and to anticipate the risk of cardiac morbidity or mortality.

## Abbreviations

ASA: American Society of Anesthesiology; CPB: cardiopulmonary bye-pass; ECG: electrocardiogram; FiO_2_: fractional inspired oxygen; ICU: Intensive Care Unit; IPPV: intermittent positive pressure ventilation; IV: intravenous; NYHA: New York Heart Association; PCV: packed cell volume; RPP: rate pressure product.

## Consent

Written informed consent was obtained from the patient for publication of this case report and accompanying images. A copy of the written consent is available for review by the Editor-in-Chief of this journal.

## Competing interests

The authors declare that they have no competing interests.

## Authors' contributions

AOJ and PA were the anaesthetists while SUO and GAA were the surgeons in attendance at surgery. All authors made contributions in the write-up of the manuscript. All authors read and approved the final manuscript.
